# Local Analgesia in the Treatment of Ano-Rectal Disease

**Published:** 1905-09

**Authors:** 


### ABSTRACTS.

### Local Analgesia in the Treatment of Ano-rectal Disease.

J. Rawson Pennington (*Journal of the Amer. Med. Asso.*, April 8, 1905,) says he has discontinued the use of cocain, finding that in many cases the toxic effects were alarming. He has used sterile water anesthesia, as suggested by Gant; but the injection causes considerable discomfort and even pain in some cases, especially those of acutely inflamed thrombotic piles and abscesses. Normal salt solution, being isotonic, is more satisfactory, the salt preventing irritative symptoms. But when injection must be made into the deeper tissues or the operation is prolonged he uses the following solution:

| | | | |
|---|---|---|---|
| ℞ | Beta-eucain lactate | 3 | grains. |
| | Sodium chloride | 11½ | " |
| | Adrenalin chloride solution | 10 | minims. |
| | Distilled water | 3½ | ounces. |

According to Barker, of London, who has made valuable contributions to the literature, the entire quantity can be used. He injected as much as 6 grains beta-eucain hydrochlorate and 20 minims adrenalin chloride solution without noting ill effects. Matas, of New Orleans, and others used larger amounts. To make the solution, put into an Erlenmayer or Jena glass flask 3½ ozs. distilled water and add 11½ grains of chemically pure sodium chloride and 3 grains beta-eucain lactate. Boil for 2 or 3 minutes; when cooled to body temperature, add 10 drops adrenalin. The author used this in radical operations for protruding and nonprotruding hemorrhoids; interno-external thrombotic and cutaneous hemorrhoids; polypi and prolapse, fistula, fissure, ulceration, abscesses, sacral dermoid, lipomas, condylomates and secondary operations for colostomy.

Since experience demonstrated that effective and radical treatment can be accomplished in the office or in the patient's home, without his going to a hospital, taking an anesthetic, and remaining there for two or three weeks, the author believes that another forward stride has been made in the treatment of selected proctological cases.

